# Development of a Microfluidic Chip System with Giant Magnetoresistance Sensor for High-Sensitivity Detection of Magnetic Nanoparticles in Biomedical Applications

**DOI:** 10.3390/bios13080807

**Published:** 2023-08-11

**Authors:** Tzong-Rong Ger, Pei-Sheng Wu, Wei-Jie Wang, Chiung-An Chen, Patricia Angela R. Abu, Shih-Lun Chen

**Affiliations:** 1 Department of Biomedical Engineering, Chung Yuan Christian University, Chung-Li 320314, Taiwan; 2 Division of Nephrology, Department of Internal Medicine, Taoyuan General Hospital, Ministry of Health and Welfare, Taoyuan 115204, Taiwan; 3 Department of Electrical Engineering, Ming Chi University of Technology, New Taipei City 243303, Taiwan; 4 Department of Information Systems and Computer Science, Ateneo de Manila University, Quezon City 1108, Philippines; 5 Department of Electronic Engineering, Chung Yuan Christian University, Taoyuan City 320314, Taiwan; chrischen@cycu.edu.tw

**Keywords:** magnetic particles, magnetoresistive sensors, microfluidics

## Abstract

Magnetic nanoparticles (MNPs) have been widely utilized in the biomedical field for numerous years, offering several advantages such as exceptional biocompatibility and diverse applications in biology. However, the existing methods for quantifying magnetic labeled sample assays are scarce. This research presents a novel approach by developing a microfluidic chip system embedded with a giant magnetoresistance (GMR) sensor. The system successfully detects low concentrations of MNPs with magnetic particle velocities of 20 mm/s. The stray field generated by the magnetic subject flowing through the microchannel above the GMR sensor causes variations in the signals. The sensor’s output signals are appropriately amplified, filtered, and processed to provide valuable indications. The integration of the GMR microfluidic chip system demonstrates notable attributes, including affordability, speed, and user-friendly operation. Moreover, it exhibits a high detection sensitivity of 10 μg/μL for MNPs, achieved through optimizing the vertical magnetic field to 100 Oe and the horizontal magnetic field to 2 Oe. Additionally, the study examines magnetic labeled RAW264.7 cells. This quantitative detection of magnetic nanoparticles can have applications in DNA concentration detection, protein concentration detection, and other promising areas of research.

## 1. Introduction

Magnetic nanoparticles (MNPs) have found widespread applications in various fields, including MRI contrast agents, magnetic recording devices, bio-sensing, drug delivery, thermal therapy, and biomolecule separation [1,2,3,4,5,6]. However, the use of large quantities of MNPs for cell manipulation in these techniques can lead to nanotoxicity concerns. Current cell assay methods for evaluating target quantities often involve techniques such as Prussian blue staining [7], T2 relaxometry [8], and UV/VIS spectrometry [9,10]. However, these approaches are primarily limited to the static detection of stray fields from immobilized labels. To address this limitation, researchers have explored the use of magnetic sensors in microfluidic applications to detect small biological samples. One such approach involves the use of a magnetic immunoassay, a novel type of immunoassay that enables the quantitative detection of biomolecules. The number of biomolecular targets, such as DNA or cells, can be determined by measuring the magnetic subject, which can be achieved through techniques such as measuring the remnant magnetic flux [11], magnetization relaxation time of magnetic particle clusters [12], or reduction in alternating current (AC) magnetic susceptibility [13]. In the past decade, magnetoresistive (MR) sensors have been utilized in magnetic biosensing to estimate the amount of target biomolecules or cells. These MR-based biosensors measure variations in MR signals caused by magnetic microparticles or nanoparticles attached to the target sample [14,15,16]. The first significant contributions to the field of detecting magnetic markers using measurements of resistance/impedance change in magnetic field sensors, including measurements in a continuous flow, can be attributed to pioneering studies. David R. Baselt et al. developed a biosensor capable of measuring the forces that bind DNA–DNA, antibody–antigen, or ligand–receptor pairs at the single-molecule level. Known as the Bead Array Counter (BARC), this biosensor utilizes interaction forces to immobilize magnetic microbeads on a solid substrate. Microfabricated magnetoresistive transducers on the substrate then indicate whether the beads are displaced when subjected to magnetic forces [17]. G. V. Kurlyandskaya et al. employed a commercial Ferrofluid^®^ liquid thin layer to cover the ribbon surface of their sensor. This innovative approach revealed that the magnetoimpedance response was significantly affected by the presence of the magnetic Ferro liquid, the applied magnetic field’s intensity, and the driving current parameters. The proposed magnetoimpedance-based prototype demonstrated high sensitivity to the fringe field generated by magnetic nanoparticles, thereby offering great promise as a biosensor [18]. F. Blanc-Béguin et al. conducted research to determine the optimal conditions for producing cell samples suitable for imaging with the detection of modifications in the magnetic field caused by maghemite (Fe_2_O_3_) nanoparticles. These nanoparticles acted as a high-sensitivity magnetic biosensor based on the giant magnetoimpedance (GMI) effect. The preliminary results from this study provided valuable insights into the production of biological samples, laying the groundwork for further advancements in GMI biosensor technology [19]. A. García-Arribas et al. introduced a microfluidic device capable of determining the concentration of magnetic beads under a continuous flow of the carrier fluid, utilizing the giant magnetoimpedance effect (GMI) [20].

However, research has demonstrated that the sensor exhibited exceptional sensitivity to liquid and materials present in the microfluidic chamber. Although it successfully detected magnetic microparticles in a static regime and magnetic nanoparticles under a continuous flow, the measurements proved to be delicate and challenging to simple measurement and analysis devices. Further research and development are required to address these intricacies and enhance the sensor’s reproducibility. In this research, the measurement systems for studying the stray fields from MNPs in MR-based biosensors rely on lock-in amplifier detection. We propose an integrated GMR microfluidic chip system as a cost-effective alternative to lock-in amplifiers. This system offers the advantages of being fast and easy to operate, as well as significantly reducing the sensing instrument costs.

## 2. Materials and Methods

**Figure 1:**
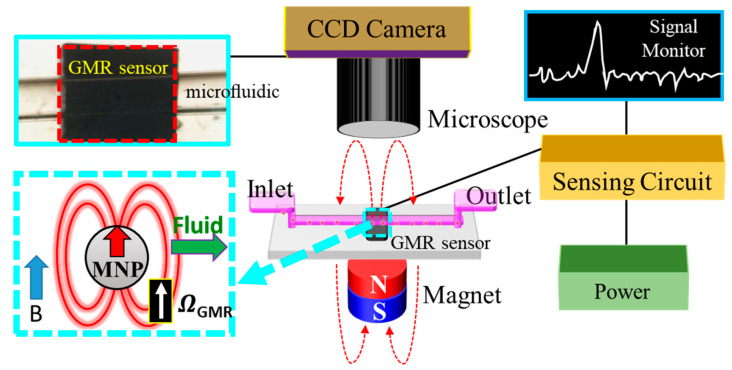
Schematic diagram of the integrated GMR microfluidic chip system, including the solution pump, detection area, and display device. The CCD camera monitors the flow of MNPs. The middle section represents the microfluidic channel. The lower part features a permanent magnet for magnetizing the superparamagnetic MNPs. The upper left inset shows the real image of the GMR microfluidic chip, while the lower left inset illustrates the MNP under the applied magnetic field (blue arrow), resulting in magnetization in the same direction (red arrow). The white arrow indicates the direction of the magnetization of the GMR, and the green arrow indicates the direction of the microfluidic flow.

The GMR general structure consisted of two ferromagnetic layers with a nonmagnetic layer sandwiched. The principle of GMR is shown in Figure 1 (red square) when the magnetization of two ferromagnetic layers parallel in the same direction and the spin electron passed through the layer with the same magnetization direction. The spin electron had a lower possibility of scattering, leading to lower magnetic resistance. On the other hand, when the two antiparallel ferromagnetic layers were magnetized, the spin electron had a higher possibility of scattering, leading to higher resistance [21]. Figure 1 is a schematic diagram of the integration of the GMR microfluidic chip. A Charge Coupled Device (CCD) camera was used to monitor the flow of MNPs, the microfluidic channel in which the MNPs flowed from the inlet to the outlet, and the permanent magnet for magnetizing the superparamagnetic MNPs. The upper blue square is the real image of the GMR microfluidic chip; the lower blue square is the MNP subjected to the applied magnetic field (blue arrow), which produced the magnetization in the same direction (red arrow). The white arrow indicates the direction of the magnetization of GMR, and the green arrow indicates the direction of the microfluidic flow. On the right is a simple diagram of signal processing. This experiment process was divided into three parts: the microfluidic channel, the synthesis of dextran-coated MNPs, and cell culture.

**Figure 2:**
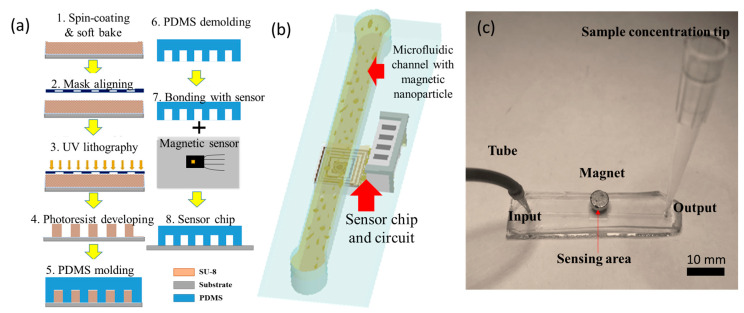
(**a**) The fabrication process of the microfluidic MNP detection chip. The lock-in MNP detection system containing the solution pump, detection area, and lock-in amplifier; (**b**) the schematic diagram of the microfluidic chip with a microchannel for magnetic nanoparticle flowing and the sensor chip and a readout circuit. (**c**) The external magnet was applied for alternative magnetization of MNPs.

The fabrication process of the microfluidic MNP detection chip shown in Figure 2a consisted of two main steps: the fabrication of the microfluidic channel and the integration of the GMR sensor and microfluidic chip. As for the microchannel fabrication, negative photoresist SU-8 was patterned and developed with (1) spin coating and soft bake on a glass substrate, (2) mask aligning, and (3) a UV lithography process (4) as a mother mold for the microchannel; then, (5) Polydimethylsiloxane (PDMS) was poured onto the mold and cured. The surface of the GMR sensor was covered by the demolding PDMS layer to form the sensor chip used in this study, and then oxygen plasma was used for the bonding between the sensor and the microchannel. Figure 2b is a schematic diagram of the microfluidic chip with a microchannel for magnetic nanoparticle flowing and the sensor chip, as well as a readout circuit for MNP detection. Figure 2c is an image of PDMS-based microfluidics with the inlet, sensing, and outlet areas. The external magnet was applied for the stable magnetization of MNPs.

**Figure 3:**
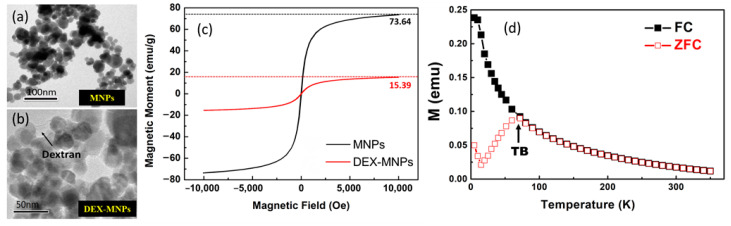
SEM image of (**a**) MNPs and (**b**) dextran-coated MNP particles. (**c**) SQUID magnetic hysteresis loops of magnetic nanoparticles (MNPs) and dextran-coated MNPs (DEX-MNPs). Magnetization ratio of DEX-MNPs and MNPs was about 0.21 (15.39/73.64). (**d**) ZFC/FC curves for MNP samples with an applied field of 100 Oe (TB = 71.82 K).

In this study, dextran-coated magnetic nanoparticles (DEX-MNPs) with an average diameter of 10 nm were synthesized and utilized. Dextran, known for its good dispersibility in aqueous solutions, was chosen as the coating material due to its widely recognized surface modification capabilities. The synthesis of DEX-MNPs followed a previously published method [22,23]. In summary, 0.405 g of iron (III) was mixed with 0.694 g of dextran in 10 mL of deionized water. The solution was injected into a 3-necked flask containing 30 mL of preheated deionized water at 80 °C under a N_2_ gas atmosphere. After 5 min of constant agitation, 0.833 mL of N_2_H_4_ was added, followed by the injection of 0.148 g of iron (II) in 10 mL of water after another 5 min. Subsequently, 8 mL of NaOH was added, and the solution was dialyzed for 24 h to remove unreacted compounds. The resulting DEX-MNPs were obtained through freeze drying. Figure 3 shows the scanning electron microscopy (SEM) images of the MNPs (Figure 3a) and DEX-MNPs (Figure 3b). The saturation magnetization of the MNPs and DEX-MNPs was measured to be 73.64 emu/g and 15.39 emu/g, respectively (Figure 3c). Figure 3d shows the magnetization curves taken in zero-field-cooling (ZFC) and field-cooling (FC) modes with an applied magnetic field of 100 Oe. The sample showed superparamagnetic behavior at room temperature, with blocking transition at TB = 71.82 K (H = 100 Oe), which is similar to results from other published papers [24,25]. For cell sample preparation, RAW 264.7 cells, from a murine macrophage cell line, were cultured in Dulbecco’s Modified Eagle’s Medium (DMEM) and supplemented with 1% penicillin and 10% fetal bovine serum (FBS) at 37 °C in a 5% CO_2_ environment. Once the cells reached 80–90% confluence in 6-well culture plates, the medium was replaced with a medium containing DEX-MNPs at a concentration of 260 µg/mL of iron, and the cells were incubated for 12 h.

**Figure 4:**
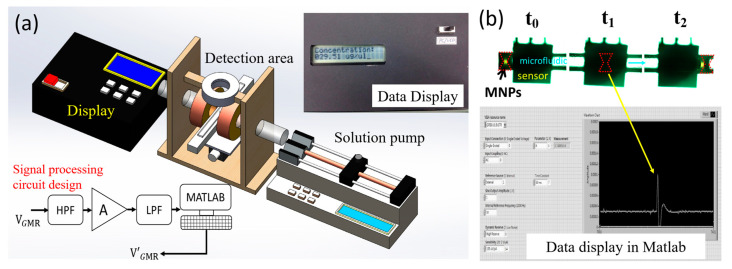
(**a**) Integration of the GMR microfluidic chip system comprising the solution pump, detection area, and data display device. The upper right inset shows the photo of the data display device. The signal processing circuit design (lower left) includes a differential amplifier, high-pass filter, low-pass filter, and amplifier with a total gain of 75 dB. (**b**) The LabVIEW software enables data synchronization from the microprocessor and display on the computer. Signal vibrations are observed on the front panel of LabVIEW when MNPs pass through the GMR microfluidic chip.

This study involved the establishment of two systems: the integration of the GMR microfluidic chip system and the lock-in MNP detection system. The GMR microfluidic chip system, illustrated in Figure 4a, comprised a solution pump, a detection area, and a data display device. The solution pump facilitated the flow of microfluidics through the channel, while the detection area consisted of microfluidic channels and GMR sensors (AAH002, NVE Corp., Eden Prairie, MN, USA) integrated with an external magnetic field for detecting the concentration of MNPs. As magnetically labeled particles passed through the GMR sensor, they induced variations in the magnetic field, leading to changes in the resistance and voltage of the sensor (ΩGMR). Thus, the concentration of MNPs could be calculated based on the signal difference. The data display device included a signal processing circuit, a microcontroller, and a display. The signal processing circuit incorporated a differential amplifier, a high-pass filter, and a low-pass filter, with high-pass and low-pass cutoff frequencies set at 5 Hz and 15 Hz, respectively, each tailored for different time spans and shapes. During the measurement of particle concentration in the flow, the sensor signals exhibited a range between 0.01 and 0.1 mVpp, with a total amplifier gain of 75 dB. The amplified output signal was captured by the MSP430f5529 microcontroller unit (Texas Instruments Incorporated, Dallas, TX, USA), which converted the analog signal from the processing circuit into a digital signal. The microprocessor then utilized a correlation equation between voltage and MNP concentration to convert the signal into concentration, which was presented on the LC display PVC160203P (Picvue Electronics CO., Hsinchu, Taiwan). The LabVIEW software enabled the synchronization of data from the microprocessor and displayed it on the computer, as depicted in Figure 4b. As the MNPs passed through the GMR microfluidic chip, signal vibrations were observed on the front panel of LabVIEW.

## 3. Results and Discussion

**Figure 5:**
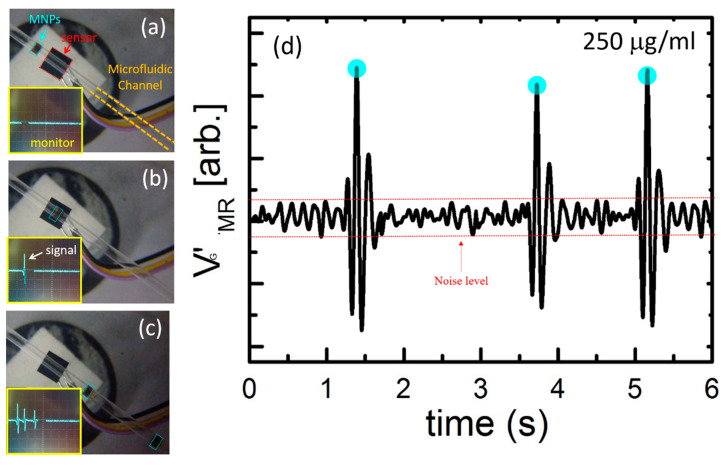
The picture of MNPs (**a**) close to the GMR sensor, (**b**) above the GMR sensor, and (**c**) pass the GMR sensor. The pictures in the lower left corner are the instant signal image. (**d**) The signal diagram of three consecutive 250 ug/mL MNPs passing through the sensor.

After confirming that the GMR sensor could detect MNPs, the GMR magnetization conditions were explored to optimize the measurement process. A preliminary detection of MNPs was conducted using an oscilloscope, which revealed that the MNPs passing through the GMR sensor caused voltage waveform vibrations, indicating the sensor’s ability to detect a small number of MNPs (Figure 5). Simultaneously, the CCD camera screened the MNPs near and above the microfluidic chip (Figure 5a,b). Figure 5c shows MNPs of the same concentration passing through the GMR microfluidic chip, while Figure 5d presents the corresponding data from the oscilloscope, demonstrating the system’s stability.

**Figure 6:**
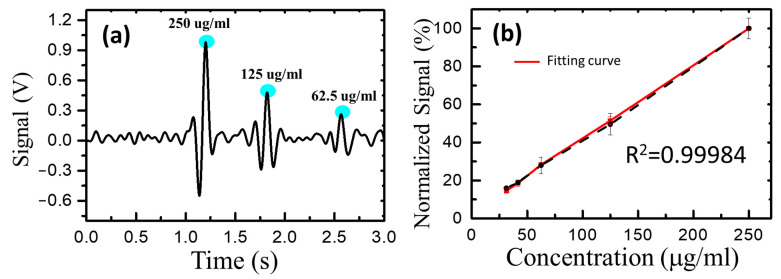
(**a**) The voltage signal of different MNP concentrations. It could be seen that, the higher the concentration of MNPs, the stronger the signal. (**b**) The linear relationship between the concentration of MNPs and the voltage. The coefficient of determination R^2^ was 0.99984.

To achieve an optimal balance between the MR sensor and the MNPs’ magnetization, an applied magnetic field was used. The vertical (*Z*-axis) magnetic field was employed to magnetize the MNPs, while the horizontal magnetic field was adjusted to find the optimal value. Experimental results indicated that a vertical magnetic field of 100 Oe and a horizontal magnetic field of 2 Oe provided the optimal measurement environment. Figure 6a displays the voltage signals of different MNP concentrations passing through the GMR microfluidic chip, illustrating a stronger signal with a higher MNP concentration. The relationship between MNP concentration and voltage showed linearity (Figure 6b) with a coefficient of determination (R^2^) of 0.99984. When examining dextran-coated MNPs with a concentration of 50 μg/μL in the integrated GMR microfluidic chip system, the measured voltage was substituted into the MNP concentration and voltage correlation function. The result indicated an MNP concentration of approximately 12 μg/μL (not shown in the figure). The magnetization ratio of dextran-coated MNPs, measured using SQUID (Figure 3c), was approximately 0.21 (15.39/73.64). This means that only 21% (10.5 μg/μL) of the MNPs in DEX-MNPs with a concentration of 50 μg/μL were magnetized, which closely aligns with the examined data.

**Figure 7:**
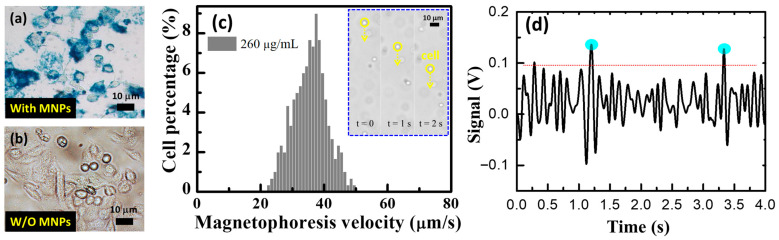
(**a**) Prussian blue staining results of RAW cells internalizing magnetic nanoparticles (MNPs) for 12 h. (**b**) Control corresponds to the RAW cells that were not treated with MPs in parallel to the treated group. (**c**) Cell velocity distributions of 510 magnetically labeled cells. Insets: consecutive optical micrographs of mobile cells at different time points. Scale bar represents 10 μm. (**d**) The measured signals responding to the magnetic-labeled RAW cells passing through the microfluidic chip system.

To examine the intracellular localizations of MNPs, cells treated with MNPs were washed three times with phosphate-buffered saline (PBS) and fixed in 4% paraformaldehyde, as depicted in Figure 7a,b. Next, a staining reagent of 2% potassium ferrocyanide with 6% HCl (1:1 *v*/*v*), known as Prussian blue stain, was added to the cells and incubated for 10 min. To quantify the MNPs, single-cell magnetophoresis was performed by subjecting the magnetic-labeled cells in suspension to a controlled magnetic field gradient. The magnetic cells suspended in the medium were exposed to the magnetic field. In the steady-state regime, the magnetic force Fm = m_bead_ dB/dx (where m_bead_ represents the magnetic moment of the magnetic beads and dB/dx is the magnetic field gradient) was balanced with the viscous force F_vis_ = 6πηRv (where R is the radius of the cell, η is the viscosity of the carrier liquid, and v is the cell velocity). The total magnetic moment of the MNPs inside a cell could be expressed as m_bead_ = NcM_s_πD^3^/6, where N is the total number of MNPs per cell, D is the diameter of an MNP, and c is the ratio of the net magnetization of the MNPs to their saturation magnetization M_s_ (set as 0.8 in this case). By setting the cell radius R and the carrier liquid viscosity η as 0.013 Pas, the number of MNPs loaded by cells N could be calculated using the following equation: N = 36ηRv/(cM_s_D^3^(dB/dX)).

A total of 510 mobile cells moving at a constant velocity toward the magnet were tracked using video microscopy. The average velocity of all the tracked cells was 35.6 ± 5.33 μm/s, as shown in Figure 7c. Applying the same method as in reference [26], the number of MNPs internalized by RAW cells was estimated to be (25.8 ± 3.86) × 10^6^. Each cell contained an average of 1.13 ng/μL of MNPs, as determined using Inductively Coupled Plasma Mass Spectrometry (ICP-MS) measurements. The magnetic-labeled RAW cells were examined using the microfluidic chip system. Figure 7d displays the measured responses corresponding to the magnetic-labeled RAW cell passing over the microfluidic chip system, indicating an MNP concentration of 30 μg/μL. This implies that each cell carried approximately 3 ng/μL, which is close to the value of 1.13 ng/μL measured using ICP-MS (not shown here). The slight difference between the values obtained from ICP-MS and our device can be attributed to operational errors and should be addressed.

## 4. Conclusions

This study successfully developed an integrated GMR microfluidic chip system for the detection of MNPs. The optimal values for the horizontal and vertical magnetic fields, determined through the lock-in MNP detection system, were found to be 2 Oe and 100 Oe, respectively. The correlation between voltage and MNP concentration was examined for both the lock-in MNP detection system and the GMR microfluidic chip system. The accuracy of both systems was validated using dextran-coated MNPs. Furthermore, the magnetic-labeled RAW cell was tested in the GMR microfluidic chip system, yielding results that closely matched those obtained using ICP-MS. This confirms that the developed integration of the GMR microfluidic chip system for MNP detection in this study offers several advantages, including a relatively low cost, fast and easy operation, and accurate measurement of magnetic particle concentration.
